# Breast Metastasis: An Unusual Cause of Malignant Breast Lesion

**DOI:** 10.5334/jbsr.3544

**Published:** 2024-03-18

**Authors:** Dunkan Petersbourg

**Affiliations:** 1 Université libre de Bruxelles, Belgium

**Keywords:** breast neoplasms, metastases – kidney neoplasms, mimic, breast carcinoma, renal cell carcinoma

## Abstract

*Teaching point:* Although rare, an intra-mammary metastasis from extramammary cancer should be considered in a patient with an oncological history.

## Case History

A 64-year-old female patient presented for her first follow-up breast examination at our institution, with no further information available on the examination request. She is postmenopausal, not using hormone replacement therapy, and reports a past breast surgery without knowing the reason.

The clinical examination revealed a scar, scar induration, and nipple retraction in the right breast’s upper quadrants; the left breast was tender, with shoulder-originating pain.

**Figure 1:**
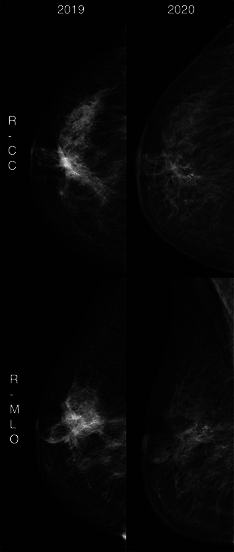
Right breast mammogram showing postsurgical architectural distortion, increased fat necrosis and calcifications.

**Figure 2:**
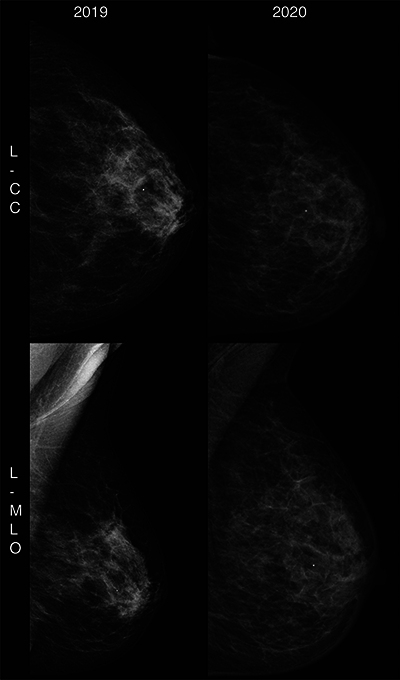
Left breast mammogram displaying diffuse skin thickening.

A mammogram was conducted, with a comparison to prior images obtained via an external platform. This revealed postsurgical architectural distortion with increased fat necrosis calcifications on the right side (Figure 1). On the left, diffuse skin thickening was observed (Figure 2).

**Figure 3:**
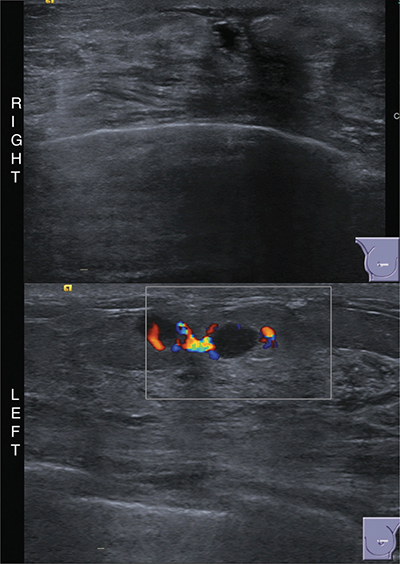
Breast ultrasound with right postsurgical changes, left reveals clustered vascularized masses.

Additionally, ultrasound imaging (Figure 3) showed postsurgical changes on the right. On the left, near the areola, there was a cluster of small masses with finely lobulated contours, slight posterior enhancement, and significant peripheral vascularization; notably, these findings were not visible on the external images. No axillary lymphadenopathy was detected.

The ultrasound findings led to a BIRADS ACR 4 classification. A biopsy revealed metastases from clear cell renal carcinoma. A review of the patient’s history uncovered bilateral clear cell renal cancer diagnosed 15 years ago, multi-metastatic, including two episodes in the right breast treated with metastasectomy. The patient is undergoing radiotherapy for metastasis to the left scapula, causing the observed skin thickening.

## Comments

Intra-mammary metastases (IMMs), originating from cancers located outside the breast, are an exceptional phenomenon, accounting for less than 1% of all malignant breast tumors. The cancers most likely to metastasize to the breast, listed in decreasing order of frequency, include melanoma, bronchopulmonary, gynecological, digestive, hematological cancers, sarcomas, and finally, renal cancers. The latter represents only 1.5% of all IMMs. IMMs commonly affect women around the age of 50, primarily in the upper outer quadrant of the breast, likely due to cancer dissemination via the bloodstream. At this age, significant vascularization of this breast region is still present before it diminishes over time [1].

Clinically, IMMs typically manifest as a round, well-demarcated, painless mass with rapid growth. Radiologically, they appear on mammography as round, well-circumscribed formations with regular to slightly irregular contours, lacking microcalcifications, except in ovarian cancer cases. On ultrasound, they present as round, hypoechoic masses without acoustic shadowing.

The clinical and radiological presentation remain nonspecific and may suggest a benign lesion, such as a fibroadenoma. The definitive diagnosis relies primarily on histopathological examination [1].

Despite their scarcity, it is crucial to recognize the possibility of an intra-mammary metastasis from an extramammary cancer in patients with a rapidly growing mass and an oncological history.
